# Understanding Older Adults’ Technology use During COVID-19 to Support Health and Connection

**DOI:** 10.1093/geroni/igaa057.3510

**Published:** 2020-12-16

**Authors:** Shani Bardach, Elizabeth Rhodus, Kelly Parsons, Allison Gibson

**Affiliations:** 1 University of Kentucky, Lexington, Kentucky, United States; 2 University of Kentucky, Richmond, Kentucky, United States; 3 University of Kentucky, College of Social Work, Lexington, Kentucky, United States

## Abstract

The COVID-19 crisis has disrupted everyday life as individuals, especially older adults, are encouraged to distance to reduce virus transmission. Remote strategies for connection may ameliorate risks for social isolation, however, older adults’ adoption of such strategies remains unknown. This study involved in-depth semi-structured interviews with 30 older adults (ages 68-94) regarding adaptations to the call for social distancing and use of technology. From a socioemotional selectivity theory lens, findings demonstrate respondents’ positive views of technology, expressing value in the context of supporting emotionally important relationships and goals, e.g. social connection and entertainment, rather than knowledge acquisition. Surprisingly, most respondents were uninterested in technology training. This may be consistent with diminished future time perspectives; several participants referenced their advanced age to explain disinterest in learning new technology and most seemed to have developed a level of technology use that met their needs. Technology resistance was consistent with a focus on emotionally meaningful goals; several respondents conveyed disinterest in social media due to perceived intrusiveness and others indicated a lack of interest in telemedicine and health portals due to the perceived loss in valuable human contact. Personal challenges with social distancing generally reflected limitations in safe human interactions, e.g. a desire for hugs or missing the spontaneity of social get-togethers, which remained emotionally meaningful but outside the scope of how technology could support wellbeing. These results suggest that focusing on older adults’ emotional goals, and highlighting how technology can support their achievement, may support meaningful use to promote health and connection.

